# Discovery of Novel Ionizable Lipids for Lipid Nanoparticles: Lipophilicity as a Predictor of Squaramide Head Group Lipid Clearance

**DOI:** 10.1002/smsc.70314

**Published:** 2026-06-01

**Authors:** E. Sathyajith Kumarasinghe, Edward J. Hennessy, Michael W. Danneman, Kristine E. Burke, Timothy Salerno, Erin E. Giardino, Matthew D. Crawford, Erin L. Thomas, Paulo L. Markaj, Farbod Mahmoudinobar, Edward Acosta, Carla Leite, Kerry E. Benenato, Juneyoung Lee, Mohindra Seepersaud

**Affiliations:** ^1^ Moderna, Inc. Cambridge Massachusetts USA

**Keywords:** cLogP, hepatic clearance, lipophilicity, protein expression, squaramide head group ionizable lipid

## Abstract

Ionizable lipids are a critical component of lipid nanoparticles (LNPs) for messenger RNA (mRNA) delivery, driving cellular uptake, endosomal escape, and tolerability. It is well‐known that chemical modifications to ionizable lipids profoundly affect both potency and safety profiles. However, the lack of reliable predictive tools, limited understanding of lipid behavior, and the intrinsic complexity of LNPs remain major challenges in LNP development. Among these, in vivo lipid clearance is a critical yet poorly understood factor influencing long‐term safety of mRNA loaded LNPs, particularly with respect to their accumulation. Impaired clearance leading to lipid accumulation can pose safety liabilities and contribute to long‐term toxicity. To address this, we investigated lipophilic properties of squaramide head group ionizable lipids with ester linkers as surrogates for in vivo clearance. We demonstrate that lipid clearance can be tuned by chemical modification and is predictable from simple lipophilicity calculation. To our knowledge, this represents the first computationally driven approach predicting lipid properties relevant to both efficacy and long‐term safety within the current competitive landscape.

## Graphical abstract

Ionizable lipids are critical for LNP‐mediated mRNA delivery, yet the relationship between their physicochemical properties and in vivo behavior remains poorly understood. This study demonstrates that lipophilicity of squaramide‐based ionizable lipids strongly correlates with liver clearance of the lipids in mice, enabling early prediction of lipid clearance during lipid design.

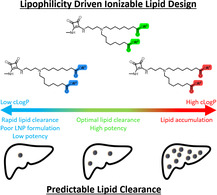

## 1 Introduction

The development of messenger RNA (mRNA)‐based therapeutics has revolutionized modern medicine, offering promising solutions for diverse diseases, including cancer, infectious diseases, and genetic disorders [1, 2, 3]. However, the clinical translation of these therapies depends on the efficient delivery of nucleic acid payloads into target cells while minimizing rapid degradation, immune activation, and suboptimal intracellular uptake [4, 5]. Lipid nanoparticles (LNPs) have emerged as the leading nonviral delivery platform for mRNA, addressing many of these challenges through their outstanding biocompatibility, optimized composition, and molecular assembly [4, 6, 7, 8]. The evolution of LNPs for gene delivery has been propelled by advances in ionizable lipid chemistry and formulation design. Initially developed for small interfering RNA (siRNA) delivery, LNPs have undergone extensive optimization to accommodate larger and more structurally complex mRNA molecules [9, 10, 11, 12, 13]. Typical LNP formulations comprise ionizable lipids, phospholipids, cholesterol, and polyethylene glycol (PEG)‐lipids, each contributing to nanoparticle stability, cellular uptake, and endosomal escape [4, 14, 15]. The overarching design rationale centers on optimizing lipid components to enhance payload protection, reduce systemic toxicity, and improve biodistribution, thereby maximizing therapeutic efficacy. LNP‐mediated mRNA delivery offers multiple advantages, including protection of mRNA from enzymatic degradation, efficient cellular uptake through endocytosis, and controlled intracellular release, collectively supporting sustained protein expression and minimizing off‐target effects [4, 16, 17]. The approval of LNP‐based mRNA vaccines against SARS‐CoV‐2 has further validated this technology and paved the way for broader applications in gene therapy and personalized medicine [18, 19].

Ionizable lipids play a central role in these systems by enabling mRNA encapsulation and intracellular delivery [10, 12, 15, 20]. Structurally, they are composed of an amine head group, a linker, and hydrophobic tails that collectively play a major role in LNP stability, biocompatibility, and transfection efficiency. Most prior research has focused on understanding structure–activity relationships (SARs) among these elements, yet predictive models linking ionizable lipid properties to biological performance remain elusive [13, 21, 22, 23, 24]. Here, we explore the correlation between in vivo lipid clearance and lipophilicity across a library of novel ionizable lipids possessing a squaramide head group. Variations in tail branching and chain length were introduced to systematically tune lipophilicity and evaluate its impact on clearance behavior. Lipophilicity was quantified using calculated LogP (cLogP) values, obtained using available commercial software. The cLogP value quantifies a computationally derived parameter that predicts the octanol‐water partition coefficient, where lower values correspond to more hydrophilic molecules and higher values indicate greater lipophilicity [25, 26]. Lipophilicity is a critical physicochemical attribute that fundamentally influences the clearance of small‐molecule drugs. Compounds with higher lipophilicity often show increased tissue distribution and stronger binding to lipophilic compartments (such as membranes and adipose tissue), which can alter hepatic uptake, shift metabolic pathways, and thereby impact clearance [27, 28, 29]. Moreover, lipophilicity has shown to relate to metabolic and excretory pathways, underpinning its value in structure–pharmacokinetic relationships [30, 31]. Despite extensive studies on the structural optimization of ionizable lipids for LNP potency and tolerability, quantitative relationships between physicochemical parameters of ionizable lipids and in vivo behavior of LNPs remain poorly defined. While prior work has focused on SARs governing delivery efficiency, endosomal escape, and biodistribution, the direct correlation between lipophilicity and lipid clearance has not been clearly established. This gap underscores the need for a more predictive framework linking molecular properties to pharmacokinetic outcomes, an area our present study seeks to address.

## 2 Results and Discussion

**FIGURE 1:**
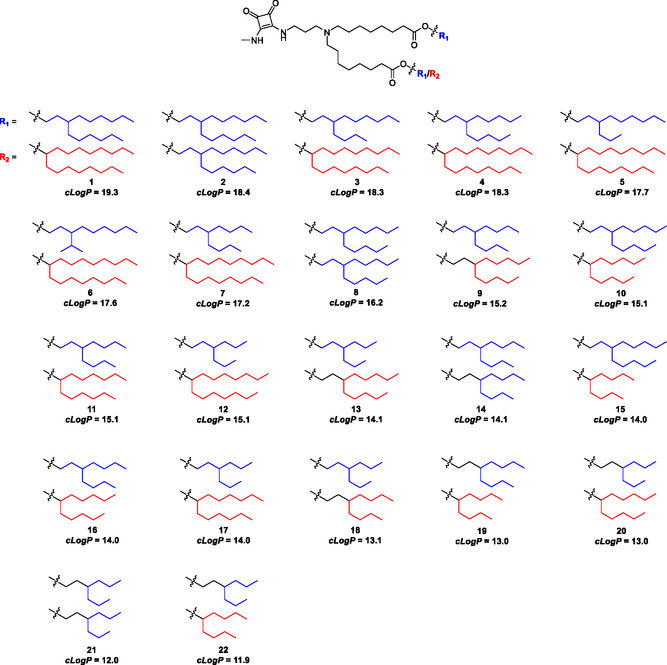
Chemical structure of prepared ionizable lipids, **1–22**, with cLogP values between 11.9 and 19.3.

**FIGURE 2:**
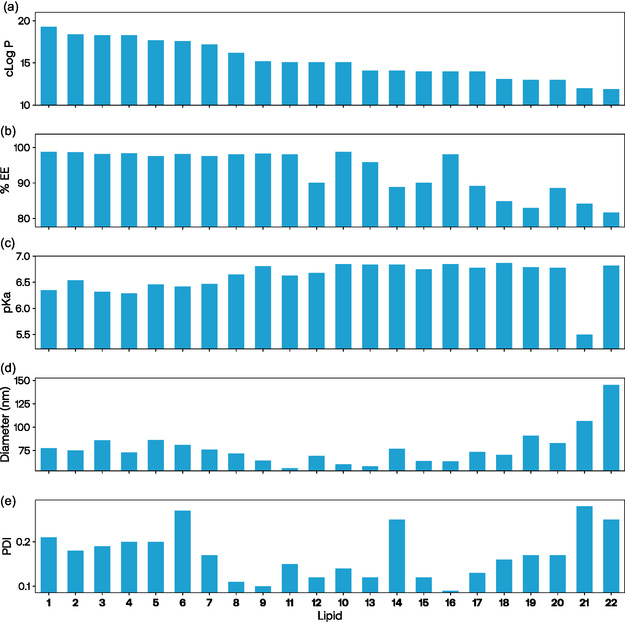
(a) cLogP of the prepared ionizable lipids, and biophysical properties, (b) %EE, (c) apparent pKa, (d) diameter, and (e) PDI of the LNPs formulated by the corresponding lipids.

To test our hypothesis that in vivo lipid clearance is correlated to lipophilicity, we synthesized 22 novel ionizable lipids containing a squaramide head group, a novel head group previously reported by our group with improved in vivo protein expression, and ester linkers to hydrophobic tails [24]. To systematically modulate lipophilicity, represented by cLogP values, α‐ and/or γ‐substituted hydrocarbon tails of varying lengths were introduced, yielding a library of ionizable lipids with cLogP values ranging from 11.9 to 19.3, encompassing both lower and higher lipophilicity levels relative to previously synthesized squaramide head group lipids with verified potency (Figure 1, Scheme S1) [24]. The cLogP values were computationally derived from ChemDraw Professional 21.0.0.28 software and can be easily obtained prior to synthesis, providing a practical parameter for early‐stage lipid design. We note that different software may produce slightly different absolute cLogP values for the same molecule. However, the relative trends between molecules are generally consistent across methods, with no reversal in rank order observed. ChemDraw was selected because it is the most widely used and readily available software among synthetic chemists. The lipids were subsequently formulated into LNPs encapsulating human erythropoietin (hEPO) mRNA. All formulated LNPs exhibited physicochemical characteristics, including particle diameter, polydispersity index (PDI), encapsulation efficiency (%EE), and apparent pKa within biologically relevant ranges (Figure 2, Table S1). Consistent with our prior work, all LNPs containing ionizable lipids with squaramide head group exhibited high mRNA encapsulation (>80%). However, varying lipophilicity of the hydrophobic tails significantly influenced formulation quality. LNPs prepared from lipids with cLogP values below 14.0, specifically **19** through **22**, generally showed less optimal %EE (<90%) and increased particle diameters. Notably, the least lipophilic lipids (**21** and **22**) formed particles exceeding 100 nm in diameter, with one reaching 145 nm, nearly twice the size of the other formulations. The combination of reduced encapsulation efficiency and enlarged particle size indicates that insufficient lipophilicity compromises LNP self‐assembly, likely due to weaker hydrophobic interactions and less compact core formation. With the squaramide head group and rest of the LNP components fixed across the series, these results strongly suggest that variations in tail lipophilicity were primarily responsible for the observed differences in formulation behavior. The pKa values were consistent across the library in the range of 6.3–6.9 except for **21**, which was a notable outlier with a pKa of 5.50. Interestingly, **21** possesses symmetric **R_1_** and **R_2_** groups (Figure 1) with the shortest hydrocarbon tails among the series. Despite having a slightly higher cLogP than **22**, LNPs formulated with **21** exhibited the lowest apparent pKa, suggesting that factors beyond bulk lipophilicity, such as molecular symmetry, chain length, and local microenvironment can significantly influence ionization behavior. Shorter, more compact hydrophobic tails may affect head group orientation or packing density within the lipid assembly, thereby reducing the apparent protonation potential of the amine. Together, these findings illustrate how subtle variations in molecular architecture beyond head group shape critical formulation parameters and may ultimately impact both potency and in vivo pharmacokinetics.

**FIGURE 3:**
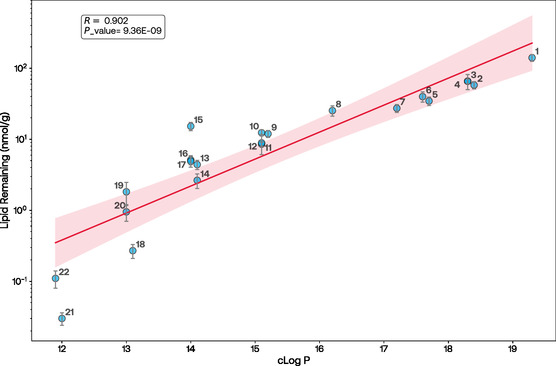
Amount of Lipid remaining (nmol/g) in CD‐1 mice liver 24 h post dose as function of cLogP for mRNA‐encapsulated LNPs. R value represents the Pearson correlation coefficient, and P_value (9.36 × 10^−9^) showing strong and significant linear relationship between cLogP and lipid clearance. 95% confidence interval band around the regression line is shown in red. The error bars represent the standard deviation in measurements from *n* = 5 animal groups. Each lipid was tested in five biological replicates (mice) for lipid remaining and data represent the mean and standard deviation for each lipid. The formulation is kept constant for all lipid groups.

The 22 LNP formulations were subsequently evaluated in vivo using CD‐1 mice models to assess hepatic retention of the lipids by quantifying residual lipid levels in the liver 24 h post intravenous (IV) administration. Remarkably, a strong linear correlation was observed between 24 h hepatic lipid retention and the cLogP of the ionizable lipids (Figure 3, Table S2). Among the series, **1**, the most lipophilic compound, exhibited the highest residual concentration in liver tissue, whereas **21** and **22**, which have the lowest cLogP values, were cleared most rapidly, approaching the detection limit of the assay. The remaining lipids in the series generally followed this trend, reinforcing the conclusion that hepatic retention is closely predicted by the lipophilicity of the tail groups in ionizable lipids. This correlation suggests that lipid lipophilicity strongly influences interactions with biological membranes, plasma proteins, and metabolic enzymes, all of which collectively determine in vivo clearance behavior. Highly lipophilic lipids likely partition more readily into hepatocyte membranes or intracellular compartments, slowing metabolic degradation, whereas less lipophilic analogs remain more accessible to hydrophilic enzymatic environments, promoting faster clearance.

In designing the lipid library, we combined hydrocarbon chains of varying lengths with both symmetric and asymmetric branching patterns and incorporated α‐ and γ‐substituted alkyl tails. This approach yielded a diverse set of ionizable lipids spanning a broad range of cLogP values, chain lengths, degrees of symmetry, and branching positions, enabling a systematic evaluation of structure–clearance relationships. We hypothesized that shifting the branching point further from the ester moiety through γ‐substitution could enhance enzymatic hydrolysis by reducing steric hindrance near the ester linkage. Among these, **1**, **4**, and **7** each possessed the same symmetric α‐substituted tail containing eight carbons but differed in the length of their symmetric γ‐substituted tails. Shorter γ‐substituted chains correlated with faster lipid clearance, with tail lengths of six (**1**), five (**4**), and four (**7**) carbons (Figure 1) resulting in 141, 66, and 40 nmol lipid per gram of liver (referred to as nmol/g in this study) of the parent lipid remaining in the liver, respectively (Figure 3, Table S2). This systematic decrease highlights that even subtle reductions in tail length can meaningfully enhance clearance, possibly by improving lipid mobility within membranes or increasing accessibility to hepatic esterases. Conversely, **4**, **10**, and **15** shared a common symmetric five‐carbon γ‐substituted tail but differed in their α‐substituted tails. In this set, lipids with shorter α‐substituted tails and correspondingly lower cLogP values exhibited more rapid hepatic clearance in 24 h period, demonstrating that lipophilicity remains the dominant factor governing clearance when branching position is held constant.

With regard to tail symmetry, **1**, **3**, **4**, **5**, **6**, and **7** all share the same α‐substituted tail (Figure 1). However, while **1**, **4**, and **7** possess symmetric γ‐substituted tails, **3**, **5**, and **6** contain asymmetric γ‐substituted tails, as illustrated in Figure 1. Regardless of symmetry, both groups followed the same trend: lipids with higher lipophilicity (higher cLogP values) exhibited slower hepatic clearance (Figure 3, Table S2). This observation reaffirms that overall hydrophobicity exerts a stronger influence on clearance than subtle geometric differences in tail symmetry and branching.

To further evaluate steric effects, **9** through **12** were analyzed as they displayed nearly identical lipophilicity (cLog*P* = 15.1–15.2), with **9** being the only compound containing two γ‐substituted tails. Based on the hypothesis that greater esterase accessibility enhances clearance, **9** was expected to show faster elimination. Contrary to this expectation, all four lipids demonstrated comparable hepatic clearance. While more focused library design and experiments are needed, this observation may suggest that within highly lipophilic compounds, steric hindrance becomes a secondary factor masked by dominant hydrophobic interactions and may reduce enzyme accessibility. A similar pattern was observed for **13–17** (cLog*P *= 14.0–14.1), where the presence of an additional γ‐substituted tail did not noticeably influence clearance. Interestingly, at lower cLogP values, such as **18–20** (cLog*P *= 13.0–13.1) and **21–22** (cLog*P *= 11.9–12.0), the γ‐substituted analogs (**18** and **21**) exhibited faster clearance than their counterparts. This suggests that steric effects may become more pronounced in less lipophilic lipids, where a more hydrophilic microenvironment may allow for better access of esterases to the lipid backbone. However, an additional lipid library with targeted experiments would be required to draw definitive conclusions regarding the effects of steric hindrance and esterase accessibility.

Collectively, these findings indicate that lipophilicity of squaramide head group ionizable lipids with ester linkages is strongly correlated with 24 h hepatic retention and represents an important design parameter, even within the context of complex LNP formulations. Although structural features such as chain branching and substitution patterns can synergistically influence metabolic accessibility, lipid lipophilicity remains the dominant factor governing in vivo clearance. Understanding this interplay provides a foundation for rational lipid design, where lipophilicity can be intentionally tuned to modulate clearance rates, enabling the development of ionizable lipids optimized for desired pharmacokinetic and safety profiles.

**FIGURE 4:**
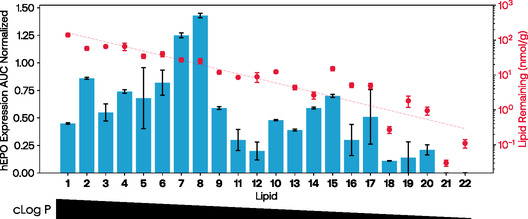
hEPO expression AUC over time (bar graph, normalized to Lipid A^*^) over 24 h and amount of lipid remaining 24h post dosage in nmol/g on alternate axis for LNPs formulated by **1–22**. Each lipid was tested in 5 biological replicates (mice) for expression and lipid remaining. Bar plots represent mean value ± std, and lipid remaining is summarized as mean ± std value for each lipid. Error bars represent the standard deviation of 5 animals per group. *Lipid A was synthesized following the literature (compound **29** in the literature) and used as a standard [24].

As demonstrated above, the cLogP of squaramide‐based ionizable lipids is a key determinant of lipid clearance. However, for these lipids to serve as effective therapeutic components, the resulting LNPs must also exhibit robust protein expression potency. To evaluate this, we assessed the in vivo expression of hEPO mRNA delivered by each of the 22 LNP formulations in CD‐1 mice models (Figure 4, Figure S1, Table S2). Analysis of in vivo hEPO expression revealed a clear relationship between lipid lipophilicity and LNP potency. In general, LNPs formulated from lipids with moderate cLogP values (≈16–17.5, **7** and **8**) achieved the highest hEPO expression Area Under the Curve (AUC) values over time, whereas both highly lipophilic (cLog*P *> 18, **1–3**) and less lipophilic (cLog*P *< 14, **18–22**) lipids resulted in markedly reduced protein expression.

The sharp decline in potency among low cLogP lipids can be partially attributed to poor formulation characteristics, including increased particle size and reduced encapsulation efficiency, which likely compromise mRNA protection and delivery. However, formulation effects alone cannot fully explain the observed differences. Low‐lipophilicity lipids may form less cohesive hydrophobic cores, weakening the stability of the nanoparticle and limiting its ability to protect and release mRNA effectively. Moreover, their decreased lipophilicity could reduce affinity for endosomal membranes, impairing endosomal escape, a critical determinant of cytosolic mRNA delivery. On the other hand, overly lipophilic lipids may integrate too strongly into endosomal or cellular membranes or form overly stable LNPs, hindering timely release of the mRNA payload into the cytoplasm and resulting in diminished translation efficiency.

Interestingly, the optimal lipophilicity range that yielded the highest hEPO expression (cLogP ≈ 16−17.5) also corresponded to an intermediate clearance rate observed in vivo. This suggests a delicate balance between metabolic stability and bioavailability: lipids that clear too slowly may accumulate in membranes and induce delayed release kinetics, while those that clear too rapidly may fail to maintain sufficient intracellular availability for efficient mRNA translation. Thus, lipophilicity emerges as a unifying parameter influencing both pharmacokinetic and pharmacodynamic behavior in LNP systems.

**FIGURE 5:**
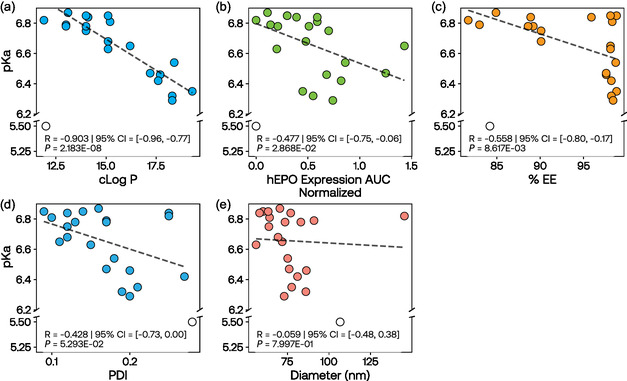
Plots between pKa and (a) cLogP, (b) hEPO expression AUC after 24hr normalized to **Lipid A**, (c) %EE, (d) PDI, and (e) diameter of the LNPs. The plots were calculated excluding a major outlier, **21** (unfilled circle). R values represent the Pearson correlation coefficient for each plot. The strongest association was observed among pKa and cLogP with Pearson R values of −0.903, with narrow confidence interval excluding zero and small *p*‐values. Panel (b), (c), and (d) showed a negative association (R = −0.477, R = −0.558, R = −0.428 and all with small P_values) between pKa and Normalized hEPO expression AUC, %EE, and PDI. These parameters have a wider confidence interval compared to cLogP, indicating greater uncertainty. Panel (e) showed no significant correlation among the pKa and Diameter.

While our primary focus is on the relationship between cLogP and lipid clearance, we also examined potential correlations between other LNP properties. Figure 5 shows cLogP, hEPO expression AUC, %EE, PDI, and particle diameter, plotted against the apparent pKa of their respective LNPs. Lipid **21** (unfilled circle) was excluded from correlation analyses due to its pronounced outlier pKa value. Among the variables examined, a strong negative correlation was observed between pKa and cLogP, indicating that lipids with higher lipophilicity tend to have lower apparent pKa values. However, no clear correlations were found between pKa and other formulation characteristics, such as hEPO expression AUC, %EE, PDI, or particle diameter. While we only evaluated apparent pKa of the LNPs, further exploration of SARs and structure–property relationships (SPRs) to identify additional, easily obtainable parameters that can predict LNP behavior or potency would be highly valuable to the field. Additionally, due to the complex nature of LNP structures and their biological pathways, artificial intelligence (AI) and machine learning (ML)‐based computational modeling would be valuable for identifying practical SAR and SPR trends through effective multivariable analysis in ionizable lipid development [32, 33]. Notably, while the prepared LNPs exhibited a range of biophysical properties (%EE, PDI, and diameter), as shown in Figures 2 and 5, lipid retention in the liver followed the trend of cLogP, with higher cLogP values associated with increased retention and lower cLogP values corresponding to reduced retention at 24 h post‐dose, regardless of the other properties evaluated.

Even though further studies are needed to delineate the precise mechanisms underlying these relationships, the present results clearly indicate that an optimal lipophilicity window is required to balance LNP stability, delivery efficiency, and metabolic clearance, ultimately maximizing mRNA translation in vivo. These findings highlight the multifaceted role of lipophilicity in LNP performance beyond clearance and formulation stability. Future studies integrating high‐throughput physicochemical modeling, lipid metabolism assays, and cellular trafficking analyses will be essential to establish predictive design rules linking lipophilicity, clearance, and mRNA expression efficiency across diverse ionizable lipid scaffolds. Collectively, these insights point toward lipophilicity‐guided rational design as a powerful strategy for next‐generation LNP optimization and will be critical for advancing predictive ionizable lipid design.

## 3 Conclusion

In summary, by only modifying the tail branching and chain lengths while keeping the rest of the ionizable lipid and other LNP components consistent, we observed a clear correlation between the lipophilicity of squaramide‐based ionizable lipids and their persistence in mouse liver following IV administration, and cLogP, a simple measure of lipophilicity that can be readily obtained from widely available chemical software, can serve as a practical predictive tool for hepatic clearance rate. While further experiments are needed, conceptually, more lipophilic lipids are likely to associate strongly with cellular and organelle membranes and bind nonspecifically to hydrophobic tissue components, thereby reducing accessibility to esterases and other metabolic enzymes. Consistent with this hypothesis, lipids bearing more lipophilic tails exhibited greater metabolic stability in vivo, whereas less lipophilic analogs exhibited more rapid clearance in the first 24 h period. These findings demonstrate that lipid clearance rates can be rationally modulated through molecular tuning of lipophilicity, offering a physicochemical handle to control the pharmacokinetic behavior of ionizable lipids. In addition, lipophilicity has a pronounced effect on LNP formulation properties. Ionizable lipids with lower cLogP values produced LNPs with larger particle diameters, higher PDIs, and reduced %EEs, indicative of compromised assembly and weakened hydrophobic packing within the nanoparticle core. These results are consistent with a recent all‐atom molecular dynamics (MD) simulation study, which showed that ionizable lipids with shorter hydrophobic tails exhibit reduced bilayer compatibility, leading to less stable LNP membrane incorporation [33]. Lipids with lower lipophilicity (e.g., shorter tails) are less compatible with stable bilayer organization and are more likely to adopt dynamic or surface‐associated states rather than remaining stably embedded. Such poor formulation characteristics translate into reduced in vivo potency, as reflected by lower hEPO expression levels. However, excessively lipophilic lipids, although stable and efficiently encapsulating, may become overly persistent in biological membranes, potentially prolonging exposure and raising safety concerns. Thus, striking an optimal balance between potency and clearance is essential for achieving both therapeutic efficacy and acceptable tolerability. We also investigated the hypothesis that steric hindrance would significantly influence lipid clearance. While we did not observe a clear trend correlating steric hindrance to clearance, when comparing α‐substituted and γ‐substituted tails across different cLogP values, our results implied that this effect is most pronounced in less lipophilic lipids, where a more hydrophilic environment permits greater enzymatic access to the ester bond. In contrast, in highly lipophilic lipids, hydrophobicity becomes the dominant factor governing clearance, as enzymatic access to the ester site is inherently restricted. Our results collectively position lipophilicity as a simple yet powerful early design parameter for rational lipid optimization. The ability to estimate cLogP computationally before the squaramide head group ionizable lipid synthesis provides a practical route to prioritize candidates with balanced physicochemical properties. Further studies employing ionizable lipids with various head groups beyond the squaramide scaffold, including ethanolamine (found in SM‐102 and ALC‐0315 used in Moderna's and Pfizer–BioNTech's COVID‐19 vaccines, respectively) and dimethylamino groups (found in DLin‐MC3‐DMA lipid), as well as systematic variations in linker and tail chemistries, will help determine whether the observed trends represent a generalizable principle across diverse ionizable lipid classes. Ultimately, integrating lipophilicity‐based design strategies with mechanistic understanding of lipid metabolism could accelerate the development of next‐generation LNPs that combine efficient delivery with favorable clearance and long‐ term safety profiles.

## Author Contributions

The manuscript was written through contributions of all authors. All authors have given approval to the final version of the manuscript.

## Conflicts of Interest

The study was funded by Moderna, Inc. All authors are current or former employees of Moderna, Inc., and may hold equity in the company.

## Supporting information

Supplementary Material

## Data Availability

The data that support the findings of this study are available in the supplementary material of this article.
