# Supplementary material for: Discovery of Novel Ionizable Lipids for Lipid Nanoparticles: Lipophilicity as a Predictor of Squaramide Head Group Lipid Clearance
Source: Small Sci. 2026 Jun 1;6(6):e70314. doi: 10.1002/smsc.70314 (PMC13248833; doi:10.1002/smsc.70314)
Supplement: Supplementary file 1 — Supplementary Material [file SMSC-6-e70314-s001.pdf]

Supporting Information

Discovery of Novel Ionizable Lipids for Lipid Nanoparticles: Lipophilicity as a Predictor of Squaramide Head Group Lipid Clearance

E. Sathyajith Kumarasinghe, Edward J. Hennessy, Michael W. Danneman, Kristine E. Burke, Timothy Salerno, Erin E. Giardino, Matthew D. Crawford, Erin L. Thomas, Paulo L. Markaj, Farbod Mahmoudinobar, Edward Acosta, Carla Leite, Kerry E. Benenato, Juneyoung Lee, and Mohindra Seepersaud**

AUTHOR ADDRESS. Moderna, Inc., 325 Binney St, Cambridge, MA 02142.

Table of Contents.

Materials and analytical methods

Experimental section

Scheme S1. Synthetic scheme for the synthesis of **1-22**

Representative synthesis of **21**

Analytical data of **1-22**

LNP formulation of ionizable lipids **1-22**

Table S1. cLogP of the ionizable lipids **1-22** and biophysical properties of the LNPs formulated from the corresponding lipids.

Procedure for the *in vivo* mice hEPO expression study and the *in vivo* mice liver collection for remaining lipid analysis

Figure S1. hEPO expression AUC normalized of the ionizable lipids **1-22**

Table S2. *In vivo* mice hEPO expression and lipids remaining in liver (nmol/g) after 24h

References

Materials and analytical methods

All chemicals and solvents were obtained from Sigma-Aldrich and Enamine and used as is unless noted. hEPO mRNA were prepared following the previous literature.[1] ^{1}H NMR spectra were obtained in $CDCl_3$ at 300K with Bruker Ultrashield 300 MHz. Silica gel chromatography was performed on ISCO CombiFlash Rf+ Lumen Instruments using ISCO RediSep Rf Gold Flash Cartridges (particle size: 20-40 microns). All final compounds were determined to be greater than 85% pure via analysis by reverse phase UPLC-MS (retention times, RT, in minutes) using Waters Acquity UPLC instrument with DAD and ELSD and a ZORBAX Rapid Resolution High Definition (RRHD) SBC18 LC column, 2.1 mm, 50 mm, 1.8 µm, and a gradient of 65 to 100% acetonitrile in water with 0.1% TFA over 5 minutes at 1.2 mL/min. Injection volume was 5 µL and the column temperature was 80 °C. Detection was based on electrospray ionization (ESI) in positive mode using Waters SQD mass spectrometer (Milford, MA, USA) and evaporative light scattering detector. The study on the % lipids remaining in liver was executed by Charles River Laboratories.

Experimental section

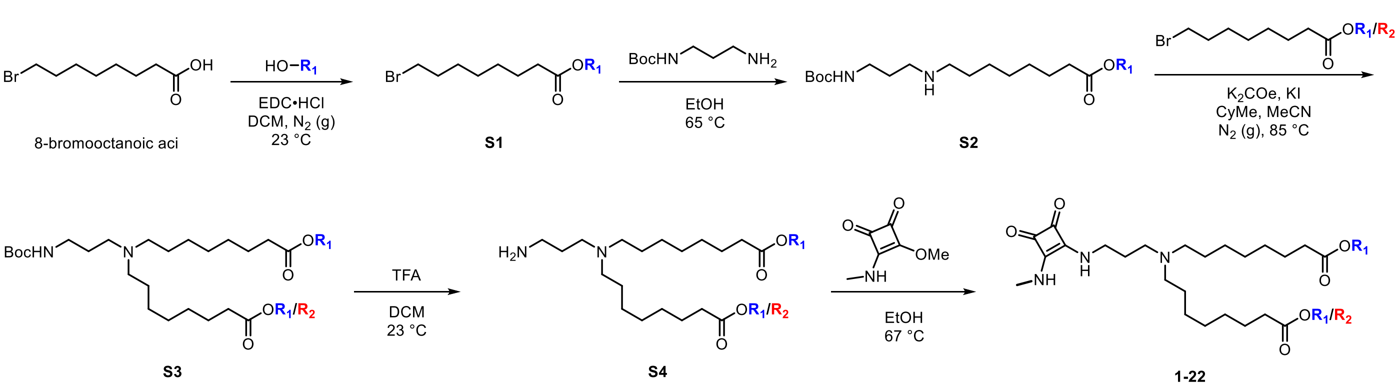

Scheme S1. Synthetic scheme for the synthesis of the ionizable lipids, **1-22**.

Representative synthesis of 21

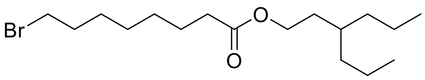

3-propylhexyl 8-bromooctanoate (S1)

To a solution of 3-propylhexan-l-ol (4.82 g, 33.4 mmol), 8-bromooctanoic acid (8.94 g, 40.1 mmol), and DMAP (816 mg, 6.68 mmol) in methylene chloride (58 mL) at 0 °C was added EDC•HCl (9.60 g, 50.1 mmol) and the reaction mixture stirred at room temperature overnight. The reaction mixture was then cooled to 0 °C and a solution of 10% aqueous hydrochloric acid (180 mL) was added slowly over 20 minutes. The layers were separated, and the organic layer was concentrated under vacuum to give a crude oil. The oil was dissolved in hexane (180 mL) and

washed with a mixture of acetonitrile (180 mL) and 5% aqueous sodium bicarbonate (180 mL). The hexane layer was separated, dried ($MgSO_4$), and filtered. The solvent was removed under vacuum to give 3-propylhexyl 8-bromooctanoate (10.9 g, 31.2 mmol, 93%) as a clear oil. The compound was carried onto the next step without further purification.
^{1}H NMR (300 MHz, $CDCl_3$) δ: ppm 4.08 (t, 2H, *J*= 6.0 Hz); 3.40 (t, 2H, *J*= 6.0 Hz); 2.28 (t, 2H, *J*= 6.0 Hz); 1.85 (pent., 2H, *J*= 6.0 Hz); 1.68-1.51 (m, 4H); 1.49-1.18 (m, 15H); 0.88 (t, 6H, *J*=6.0 Hz).

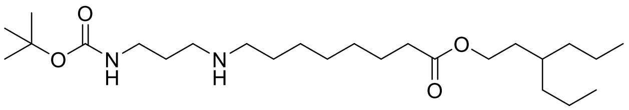

3-propylhexyl 8-((3-((*tert*-butoxycarbonyl)amino)propyl)amino)octanoate (S2)
To a solution of *tert*-butyl-(3-aminopropyl)carbamate (23.9 g, 137 mmol) in EtOH (60 mL) was added 3-propylhexyl 8-bromooctanoate (8.00 g, 22.3 mmol) in EtOH (20 mL) over the course of 20 min. The reaction was heated to 60 °C and allowed to stir at this temperature for 16 h. Upon cooling, the solvents were evaporated and the residue was diluted with ethyl acetate and washed with saturated aqueous $NaHCO_3$ and brine (5X) until no white precipitate was observed in the aqueous layer. The organic layer was separated, washed with brine, dried ($MgSO_4$), filtered, and concentrated. The residue was purified by flash chromatography (0-5-10-25-50-100% (mixture of 1% NH_4OH, 20% MeOH in dichloromethane) in dichloromethane) to give 3-propylhexyl 8-((3-((*tert*-butoxycarbonyl)amino)propyl)amino)octanoate (5.81 g, 13.1 mmol, 57%) as a clear oil.
^{1}H NMR (300 MHz, $CDCl_3$) δ: ppm 5.16 (br. s, 1H); 4.08 (t, 2H, *J*= 6.0 Hz); 3.19 (br. q, 2H, *J*= 6.0 Hz); 2.65 (t, 2H, *J*= 6.0 Hz); 2.56 (t, 2H, *J*= 6.0 Hz); 2.27 (t, 2H, *J*= 6.0 Hz); 1.70-1.51 (m, 6H); 1.50-1.39 (m, 3H); 1.43 (s, 9H); 1.36-1.17 (m, 15H); 0.88 (t, 6H, *J*= 6.0 Hz).

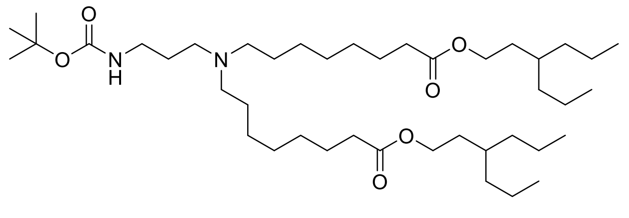

Bis(3-propylhexyl) 8,8'-((3-((*tert*-butoxycarbonyl)amino)propyl)azanediyl)dioctanoate (S3)
To a solution of 3-propylhexyl 8-bromooctanoate (735 mg, 2.11 mmol) and 3-propylhexyl 8-((3-((*tert*-butoxycarbonyl)amino)propyl)amino)octanoate (1.00 g, 2.01 mmol) in cyclopentyl methyl ether (9 mL) and actonitrile (9 mL) was added potassium carbonate (1.66 g, 12.0 mmol) and iodopotassium (366 mg, 2.21 mmol). The reaction was allowed to stir at 80 °C for 16 h. Upon cooling, the volatiles were evaporated under vacuum. The residue was diluted with dichloromethane and washed with water. The organic layer was separated, washed with brine, dried ($MgSO_4$), filtered, and concentrated. The crude residue was purified by silica gel chromatography (0-5-10-25-50-100% (mixture of 1% NH4OH, 20% MeOH in dichloromethane) in dichloromethane) to give bis(3-propylhexyl) 8,8'-((3-((*tert*-butoxycarbonyl)amino)propyl) azanediyl) dioctanoate (698 mg, 0.91 mmol, 45%) as a golden oil.

UPLC/ELSD: RT = 2.58 min. MS (ES): m/z (MH^+) 711.59 for $C_{42}H_{82}N_2O_6$.
^{1}H NMR: (300 MHz, $CDCl_3$) δ 4.08 (t, *J* = 7.1 Hz, 4H), 3.17 (d, *J* = 6.2 Hz, 2H), 2.43 (t, *J* = 6.5 Hz, 2H), 2.35 (d, *J* = 7.3 Hz, 3H), 2.32 – 2.23 (m, 5H), 1.59 (dq, *J* = 10.1, 7.3 Hz, 11H), 1.43 (s, 15H), 1.36 – 1.14 (m, 28H), 0.88 (t, *J* = 6.7 Hz, 12H).

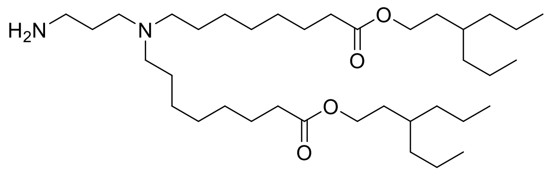

Bis(3-propylhexyl) 8,8'-((3-aminopropyl)azanediyl)dioctanoate (S4)

To a solution of bis(3-propylhexyl) 8,8'-((3-((*tert*-butoxycarbonyl)amino)propyl)azanediyl) dioctanoate (698 mg, 0.91 mmol) in methylene chloride (18 mL) was added trifluoroacetic acid (1.39 mL, 18.2 mmol). The reaction was allowed to stir at room temperature for 4 h. The reaction was quenched with saturated aqueous $NaHCO_3$ and extracted with dichloromethane. The organic layer was separated, washed with brine, dried ($MgSO_4$), filtered and concentrated. The crude material was purified by silica gel chromatography (0-5-10-25-50-100% (mixture of 1% NH_4OH, 20% MeOH in dichloromethane) in dichloromethane) to give bis(3-propylhexyl) 8,8'-((3-aminopropyl)azanediyl)dioctanoate (378 mg, 0.57 mmol, 62%) as a clear oil.
UPLC/ELSD: RT = 1.93 min. MS (ES): m/z (MH^+) 611.44 for $C_{37}H_{74}N_2O_4$.
^{1}H NMR: (300 MHz, $CDCl_3$) δ 4.08 (t, *J* = 7.1 Hz, 4H), 2.76 (t, *J* = 6.7 Hz, 2H), 2.48 (t, *J* = 7.1 Hz, 2H), 2.43 – 2.34 (m, 4H), 2.28 (t, *J* = 7.5 Hz, 6H), 1.69 – 1.51 (m, 10H), 1.50 – 1.36 (m, 7H), 1.36 – 1.16 (m, 28H), 0.88 (t, *J* = 6.7 Hz, 12H).

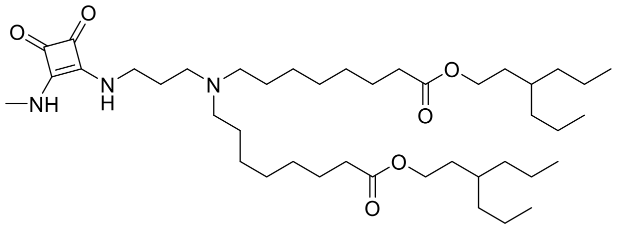

Bis(3-propylhexyl) 8,8'-((3-((2-(methylamino)-3,4-dioxocyclobut-1-en-1-yl)amino)propyl) azanediyl)dioctanoate (21)

To a solution of bis(3-propylhexyl) 8,8'-((3-aminopropyl)azanediyl)dioctanoate (378 mg, 0.57 mmol) in ethanol (5 mL) was added 3-methoxy-4-(methylamino)cyclobut-3-ene-l,2-dione (120 mg, 0.85 mmol). The reaction was allowed to stir at 67 °C for 20 h. After 20 h, the reaction was cooled to room temperature and diluted with diethyl ether. The organics were washed with brine, dried ($MgSO_4$), filtered, and concentrated. The crude residue was purified by silica gel chromatography (0-5-10-25-50-100% (mixture of 1% NH_4OH, 20% MeOH in dichloromethane) in dichloromethane) to give bis(3-propylhexyl) 8,8'-((3-((2-(methylamino)-3,4-dioxocyclobut-l-

en-l-yl)amino)propyl)azanediyl)dioctanoate (198 mg, 0.26 mmol, 45%) as light-yellow amorphous solid.
UPLC/ELSD: RT = 2.54 min. MS (ES): m/z (MH^+) 776.22 for $C_{46}H_{85}N_3O_6$.
^1H NMR (300 MHz, $CDCl_3$) δ: ppm 7.25 (br. s, 1H); 4.07 (t, 4H, *J*= 6.0 Hz); 3.65 (br. s, 2H); 3.27 (d, 3H, *J*= 6.0 Hz); 2.54 (br. t, 2H, *J*= 6.0 Hz); 2.41 (br. t, 4H, *J*= 6.0 Hz); 2.29 (t, 4H, *J*= 6.0 Hz); 1.74 (br. pent., 2H, *J*= 6.0 Hz); 1.67-1.52 (m, 8H); 1.49-1.18 (m, 43H); 0.88 (t, 12H, *J*= 6.0 Hz).

Analytical data of 1-22

Heptadecan-9-yl 8-((8-((3-hexylnonyl)oxy)-8-oxooctyl)(3-((2-(methylamino)-3,4-dioxocyclobut-1-en-1-yl)amino)propyl)amino)octanoate (1)
UPLC/ELSD: RT = 3.17 min. MS (ES): m/z (MH^+) 917.085 for $C_{58}H_{105}N_3O_6$.
^1H NMR (300 MHz, $CDCl_3$) δ: ppm 4.88 (p, 1H); 4.10 (t, 2H); 3.68 (bm, 2H); 3.28 (m; 3H); 2.75-2.44 (m, 6H); 2.31 (m, 4H); 1.82 (m, 3H); 1.72-1.18 (m, 71H); 0.90 (m, 12H).

Bis(3-hexylnonyl 8,8'-((3-((2-(methylamino)-3,4-dioxocyclobut-1-en-1-yl)amino)propyl)azanediyl)dioctanoate (2)
UPLC/ELSD: RT = 3.08 min. MS (ES): m/z (MH^+) 889.332 for $C_{54}H_{101}N_3O_6$.
^1H NMR (300 MHz, $CDCl_3$) δ: ppm 4.10 (m, 4H); 3.67 (bm, 2H); 3.28 (m, 3H); 2.57 (m, 2H); 2.44 (m, 4H); 2.31 (m, 4H); 1.77 (m, 2H); 1.62 (m, 8H); 1.52-1.19 (m, 58H); 0.90 (m, 12H).

3-Butylnonyl 8-((8-(heptadecan-9-yloxy)-8-oxooctyl)(3-((2-(methylamino)-3,4-dioxocyclobut-1-en-1-yl)amino)propyl)amino)octanoate (3)
UPLC/ELSD: RT = 3.01 min. MS (ES): m/z (MH^+) 888.7 for $C_{54}H_{101}N_3O_6$.
^1H NMR (300 MHz, $CDCl_3$) δ: ppm 5.23 (s, 1H); 4.78 (quint., 1H, *J*= 6 Hz); 4.01 (m, 2H, *J*= 5 Hz); 3.58 (br. s, 2H); 3.20 (d, 3H, *J*= 6 Hz); 2.50 (br. s, 2H); 2.37 (br. m, 4H); 2.22 (br. t, 4H); 1.69 (br.m, 5H); 1.55 (br. m, 6H); 1.43 (br. m, 5H); 1.35 (br. m, 5H); 1.19 (br. s, 50H); 0.81 (br. m, 12H).

Heptadecan-9-yl 8-((3-((2-(methylamino)-3,4-dioxocyclobut-1-en-1-yl)amino)propyl)(8-oxo-8-((3-pentyloctyl)oxy)octyl)amino)octanoate (4)
UPLC/ELSD: RT = 3.12 min. MS (ES): m/z (MH^+) 889.332 for $C_{54}H_{101}N_3O_6$.
^1H NMR (300 MHz, $CDCl_3$) δ: ppm 4.87 (p, 1H); 4.10 (t, 2H); 3.67 (bm, 2H); 3.29 (m, 3H); 2.67-2.41 (m, 6H); 2.31 (m, 4H); 1.85-1.18 (m, 71H), 0.90 (m, 12H).

Heptadecan-9-yl 8-((3-((2-(methylamino)-3,4-dioxocyclobut-1-en-1-yl)amino)propyl)((8-oxo-8-((3-propylnonyl)oxy)octyl))amino)octanoate (5)
UPLC/ELSD: RT = 2.93 min. MS (ES): m/z (MH^+) 875.5 for $C_{53}H_{99}N_3O_6$.
^1H NMR (300 MHz, $CDCl_3$) δ: ppm 5.32 (s, 2H); 4.87 (quint., 1H, *J*= 5 Hz); 4.10 (t, 2H, *J*= 8 Hz); 3.68 (br. s, 2H); 3.30 (d, 3H, *J*= 5 Hz); 2.67 (br. s, 2H); 2.53 (br. m, 4H); 2.31 (t, 5H, *J*= 7 Hz); 1.83 (br. m, 3H); 1.62 (br. m, 7H); 1.51 (br. m, 10H); 1.28 (br. s, 52H); 0.90 (br. m, 13H).

Heptadecan-9-yl 8-((8-((3-isopropylnonyl)oxy)-8-oxooctyl)(3-((2-(methylamino)-3,4-dioxocyclobut-1-en-1-yl)amino)propyl)amino)octanoate (6)
UPLC/ELSD: RT = 2.92 min. MS (ES): m/z (MH^+) 875.5 for $C_{53}H_{99}N_3O_6$.

^1H NMR (300 MHz, $CDCl_3$) δ: ppm 5.32 (s, 1H); 4.87 (quint., 1H, *J*= 6 Hz); 4.08 (m, 2H, *J*= 7 Hz); 3.67 (br. s, 2H); 3.30 (d, 3H, *J*= 5 Hz); 2.64 (br. s, 2H); 2.51 (br. m, 4H); 2.31 (m, 5H); 1.81 (br. m, 3H); 1.64 (br. m, 6H); 1.52 (br. m, 9H); 1.28 (br. s, 46H); 0.90 (br. m, 15H).

3-Butylheptyl 8-((8-(heptadecan-9-yloxy)-8-oxooctyl)(3-((2-(methylamino)-3,4-dioxocyclobut-1-en-1-yl)amino)propyl)amino)octanoate (7)

UPLC/ELSD: RT = 2.98 min. MS (ES): m/z (MH^+) 861.714 for $C_{52}H_{97}N_3O_6$.

^1H NMR (300 MHz, $CDCl_3$) δ: ppm 4.89 (p, 1H); 4.10 (t, 2H); 3.75 (m, 2H); 3.39-3.20 (m, 5H); 3.08 (m, 4H); 2.31 (m, 4H); 2.12 (bm, 2H); 1.81-1.20 (m, 65H); 0.90 (m, 12H).

Bis(3-pentyloctyl) 8,8'-((3-((2-(methylamino)-3,4-dioxocyclobut-1-en-1-yl)amino)propyl) azanediyl)dioctanoate (8)

UPLC/ELSD: RT = 2.79 min. MS (ES): m/z (MH^+) 832.34 for $C_{50}H_{93}N_3O_6$.

^1H NMR (300 MHz, $CDCl_3$) δ: ppm 7.83 (br. s, 1H); 7.61 (br. s, 1H); 4.03 (t, 4H, *J*= 9.0 Hz); 3.64 (br. s, 2H); 3.28 (br. d, 3H, *J*= 6.0 Hz); 2.46 (t, 2H, *J*= 9.0 Hz); 2.33 (br. t, 4H, *J*= 6.0 Hz); 2.33 (t, 4H, *J*= 9.0 Hz); 1.74 (br. pentet, 2H, *J*= 6.0 Hz); 1.62-1.47 (m, 8H); 1.41-1.12 (m, 50H); 0.83 (t, 12H, *J*= 9.0 Hz).

3-Butylheptyl 8-((3-((2-(methylamino)-3,4-dioxocyclobut-1-en-1-yl)amino)propyl)(8-oxo-8-((3-pentyloctyl)oxy)octyl)amino)octanoate (9)

UPLC/ELSD: RT = 2.67 min. MS (ES): m/z (MH^+) 804.22 for $C_{48}H_{89}N_3O_6$.

^1H NMR (300 MHz, $CDCl_3$) δ: ppm 7.38 (br. s, 1H); 7.03 (br. s, 1H); 4.07 (t, 4H, *J*= 6.0 Hz); 3.65 (br.s, 2H, *J*= 6.0 Hz); 3.27 (d, 3H, *J*= 6.0 Hz); 2.52 (br. t, 2H, *J*= 6.0 Hz); 2.40 (br. t, 4H, *J*= 6.0 Hz); 2.28 (t, 4H, *J*= 6.0 Hz); 1.75 (br. pent., 2H, *J*= 6.0 Hz); 1.67-1.51 (m, 8H); 1.47-1.17 (m, 46H); 0.93-0.82 (m, 12H).

3-Pentyloctyl 8-((3-((2-(methylamino)-3,4-dioxocyclobut-1-en-1-yl)amino)propyl)(8-oxo-8-(undecane-6-yloxy)octyl)amino)octanoate (10)

UPLC/ELSD: RT= 2.68 min. MS (ESI): m/z for (M^+H) 804.255 for $C_{48}H_{90}N_3O_6$.

^1H NMR (300 MHz, $CDCl_3$) δ: ppm 4.88 (p, 1H); 4.10 (t, 2H); 3.67 (br. s, 2H); 3.30 (d, 3H); 2.59 (br. t, 2H); 2.41 (br. t, 4H); 2.31(t, 4H); 1.78-1.28 (m, 58H); 0.90 (t, 12H).

3-Butylheptyl 8-((3-((2-(methylamino)-3,4-dioxocyclobut-1-en-1-yl)amino)propyl)(8-oxo-8-(tridecan-7-yloxy)octyl)amino)octanoate (11)

UPLC/ELSD: RT = 2.74 min. MS (ES): m/z (MH^+) 804.220 for $C_{48}H_{89}N_3O_6$.

^1H NMR (300 MHz, $CDCl_3$) δ: ppm 4.88 (p, 1H); 4.10 (t, 2H); 3.70 (bm, 2H); 3.30 (m, 3H); 2.90-2.57 (m, 5H); 2.31 (m, 4H); 1.90 (bm, 2H); 1.74-1.19 (m, 56H); 0.91 (m, 12H).

Pentadecan-8-yl 8-((3-((2-(methylamino)-3,4-dioxocyclobut-1-en-1-yl)amino)propyl)(8-oxo-8-((3-propylhexyl)oxy)octyl)amino)octanoate (12)

UPLC/ELSD: RT= 2.75 min. MS (ESI): m/z for (M^+H) 804.255 for $C_{48}H_{90}N_3O_6$.

^1H NMR (300 MHz, $CDCl_3$) δ: ppm 9.05 (br. s, 1H); 8.37 (br. s, 1 H); 4.88 (p, 1H); 4.10 (t, 2H); 3.73 (br. d, 2H); 3.34 (d, 3H); 3.26 (m, 2H); 3.07 (br. t, 4H); 2.30 (t, 4H); 2.11 (br. t, 2H); 1.71-1.28 (m, 61H); 0.90 (t, 12H).

3-Pentyloctyl 8-((3-((2-(methylamino)-3,4-dioxocyclobut-1-en-1-yl)amino)propyl)(8-oxo-8-((3-propylhexyl)oxy)octyl)amino)octanoate (13)
UPLC/ELSD: RT = 2.54 min. MS (ES): m/z (MH^+) 776.22 for $C_{46}H_{85}N_3O_6$.
^{1}H NMR (300 MHz, $CDCl_3$) δ: ppm 7.25 (br. s, 1H); 4.07 (t, 4H, *J*= 6.0 Hz); 3.65 (br. s, 2H); 3.27 (d, 3H, *J*= 6.0 Hz); 2.54 (br. t, 2H, *J*= 6.0 Hz); 2.41 (br. t, 4H, *J*= 6.0 Hz); 2.29 (t, 4H, *J*= 6.0 Hz); 1.74 (br. pent., 2H, *J*= 6.0 Hz); 1.67-1.52 (m, 8H); 1.49-1.18 (m, 43H); 0.88 (t, 12H, *J*= 6.0 Hz).

Bis(3-butylheptyl) 8,8'-((3-((2-(methylamino)-3,4-dioxocyclobut-1-en-1-yl)amino)propyl) azanediyl)dioctanoate (14)
UPLC/ELSD: RT = 2.52 min. MS (ES): m/z (MH^+) 776.462 for $C_{46}H_{85}N_3O_6$.
^{1}HNMR (300 MHz, $CDCl_3$) δ: ppm 4.10 (m, 4H); 3.67 (br. m, 2H); 3.29 (m, 3H); 2.58 (m, 2H); 2.43 (m, 4H); 2.32 (t, 4H); 1.84-1.16 (m, 54H); 0.91 (m, 12H).

Nonan-5-yl 8-((3-((2-(methylamino)-3,4-dioxocyclobut-1-en-1-yl)amino)propyl)(8-oxo-8-((3-pentyloctyl)oxy)octyl)amino)octanoate (15)
UPLC/ELSD: RT = 2.55 min. MS (ES): m/z (MH^+) 776.34 for $C_{46}H_{85}N_3O_6$.
^{1}H NMR (300 MHz, $CDCl_3$) δ: ppm 7.22 (br. s, 1H); 6.80 (br. s, 1H); 4.86 (pent., 1H, *J*= 6.0 Hz); 4.07 (t, 2H, *J*= 6.0 Hz); 3.65 (br. s, 2H); 3.27 (d, 3H, *J*= 6.0 Hz); 2.54 (br. t, 2H, *J*= 6.0 Hz); 2.41 (br. t, 4H, *J*= 6.0 Hz); 2.29 (t, 4H, *J*= 6.0 Hz); 1.74 (br. pent., 2H, *J*= 6.0 Hz); 1.67-1.47 (m, 10H); 1.45-1.19 (m, 41H); 0.88 (t,12H, *J*= 6.0 Hz)

3-Butylheptyl 8-((3-((2-(methylamino)-3,4-dioxocyclobut-1-en-1-yl)amino)propyl)(8-oxo-8-(undecan-6-yloxy)octyl)amino)octanoate (16)
UPLC/ELSD: RT = 2.56 min. MS (ES): m/z (MH^+) 776.344 for $C_{46}H_{85}N_3O_6$.
^{1}H NMR (300 MHz, $CDCl_3$) δ: ppm 4.88 (p, 1H); 4.10 (t, 2H); 3.72 (br. m, 2H); 3.33 (m, 3H);3.15-2.72 (m, 5H); 2.31 (m, 4H); 2.01 (bm, 2H); 1.77-1.18 (m, 52H); 0.91 (m, 12H).

3-Propylhexyl 8-((3-((2-(methylamino)-3,4-dioxocyclobut-1-en-1-yl)amino)propyl)(8-oxo-8-(tridecan-7-yloxy)octyl)amino)octanoate (17)
UPLC/ELSD: RT = 2.48 min found, MS (ES): m/z (MH^+) 776.34 for $C_{46}H_{85}N_3O_6$.
^{1}H NMR (300 MHz, $CDCl_3$) δ: ppm 4.88 (p, 1H); 4.10 (t, 2H); 3.78-3.55 (m, 2H); 3.29 (d, 3H); 2.56 (t, 2H); 2.43 (t, 4H); 2.31 (t, 4H); 1.85-1.70 (m, 2H); 1.70-1.48 (m, 11H); 1.48-1.39 (m, 4H); 1.39-1.17 (m, 36H); 0.90 (t, 12H).

3-Butylheptyl 8-((3-((2-(methylamino)-3,4-dioxocyclobut-1-en-1-yl)amino)propyl)(8-oxo-8-((3-propylhexyl)oxy)octyl)amino)octanoate (18)
UPLC/ELSD: RT = 2.43 min. MS (ES): m/z (MH^+) 748.47 for $C_{44}H_{81}N_3O_6$.
^{1}H NMR (300 MHz, $CDCl_3$) δ: ppm 7.51 (br. s, 1H); 7.19 (br. s, 1H); 4.06 (t, 4H, *J*= 6.0Hz); 3.65 (br. s, 2H); 3.28 (d, 3H, *J*= 6.0 Hz); 2.51 (br. t, 2H, *J*= 6.0 Hz); 2.39 (br. t, 4H, *J*= 6.0 Hz); 2.27 (t, 4H, *J*= 6.0 Hz); 1.75 (br. pent., 2H, *J*= 6.0 Hz); 1.67-1.51 (m, 8H); 1.47-1.16 (m, 38H); 0.92-0.82 (m, 12H).

3-Butylheptyl 8-((3-((2-(methylamino)-3,4-dioxocyclobut-1-en-1-yl)amino)propyl)(8-(nonan-5-yloxy)-8-oxooctyl)amino)octanoate (19)
UPLC/ELSD: RT = 2.36 min. MS (ES): m/z (MH^+) 748.344 for $C_{44}H_{81}N_3O_6$.

^{1}H NMR (300 MHz, $CDCl_3$) δ: ppm 4.88 (p, 1H); 4.10 (t, 2H); 3.68 (br. m, 2H); 3.30 (m, 3H); 2.74-2.43 (m, 6H); 2.31 (m, 4H); 1.82 (br. m, 2H); 1.73-1.16 (m, 47H); 0.91 (m, 12H).

3-Propylhexyl 8-((3-((2-(methylamino)-3,4-dioxocyclobut-1-en-1-yl)amino)propyl)(8-oxo-8-(undecan-6-yloxy)octyl)amino)octanoate (20)
UPLC/ELSD: RT = 2.42 min found, MS (ES): m/z (MH^+) 748.47 for $C_{44}H_{81}N_3O_6$.
^{1}H NMR (300 MHz, $CDCl_3$) δ: ppm 4.88 (p, 1H); 4.10 (t, 2H); 3.76-3.57 (m, 2H); 3.29 (d, 3H); 2.56 (t, 2H); 2.43 (t, 4H); 2.31 (t, 4H); 1.84-1.71 (m, 2H); 1.71-1.48 (m, 11H); 1.48-1.40 (m, 4H); 1.40-1.17 (m, 32H); 0.91 (t, 12H).

Nonan-5-yl 8-((3-((2-(methylamino)-3,4-dioxocyclobut-1-en-1-yl)amino)propyl)(8-oxo-8-((3-propylhexyl)oxy)octyl)amino)octanoate (22)
UPLC/ELSD: RT = 2.24 min MS (ES): m/z (MH^+) 720.22 for $C_{42}H_{77}N_3O_6$.
^{1}H NMR (300 MHz, $CDCl_3$) δ: ppm 4.88 (p, 1H); 4.10 (t, 2H); 3.77-3.55 (m, 2H); 3.29 (d, 3H); 2.56 (t, 2H); 2.43 (t, 4H); 2.31 (t, 4H); 1.89-1.70 (m, 3H); 1.70-1.50 (m, 10H); 1.50-1.39 (m, 4H); 1.39-1.17 (m, 28H); 0.91 (t, 12H).

LNP formulation of ionizable lipid 1-22

Lipid nanoparticles (LNPs) were prepared following the previous literature published from our group.[1,2] Briefly, a mixture of lipids containing ionizable lipid, phospholipid, structural lipid, and PEG lipid was dissolved in ethanol. This ethanol solution was combined with a solution of mRNA in a sodium acetate buffer through a high-efficiency mixer at a target of N:P ratio 3.4. This lipid nanoparticle solution was then concentrated and buffer exchanged into a 20 mM tris 8% sucrose pH 7.4 buffer. Formulated lipid nanoparticles were vialed and stored refrigerated at 5 °C until use. A release panel of analytical assays including particle size, polydispersity (PDI), apparent pKa, and mRNA encapsulation efficiency (%EE) was performed on all samples which were deemed acceptable for use *in vivo*.

Table S1. cLogP of the ionizable lipids **1-22** and biophysical properties of the LNPs formulated from the corresponding lipids.

Lipids	cLog P	%EE	Diameter (nm)	PDI	pKa
**1**	19.3	98.8	77.6	0.21	6.35
**2**	18.4	98.7	75.2	0.18	6.54
**3**	18.3	98.2	86.1	0.19	6.32
**4**	18.3	98.4	73.1	0.20	6.29
**5**	17.7	97.6	86.4	0.20	6.46
**6**	17.6	98.2	81.1	0.27	6.42
**7**	17.2	97.6	76.1	0.17	6.47
**8**	16.2	98.1	71.9	0.11	6.65

**9**	15.2	98.3	64.3	0.10	6.81
**10**	15.1	90.1	69.4	0.12	6.68
**11**	15.1	98.8	60.4	0.14	6.85
**12**	15.1	98.1	56.2	0.15	6.63
**13**	14.1	95.9	58.3	0.12	6.84
**14**	14.1	88.9	77.0	0.25	6.84
**15**	14.0	90.1	63.9	0.12	6.75
**16**	14.0	98.1	63.5	0.09	6.85
**17**	14.0	89.2	73.6	0.13	6.78
**18**	13.1	84.9	70.5	0.16	6.87
**19**	13.0	83.0	91.0	0.17	6.79
**20**	13.0	88.6	83.1	0.17	6.78
**21**	12.0	84.2	106.7	0.28	5.50
**22**	11.9	81.7	145.3	0.25	6.82

Procedure for the *in vivo* mice hEPO expression study and the in vivo mice liver collection for remaining lipid analysis

Specific-pathogen-free female CD-1 mice (18–22 g, n=5 per group) were obtained from Charles River Laboratories (Kingston, NY). Animals were housed in microisolator cages within a BSL-2 facility and provided food and water ad libitum. All animal studies were performed in accordance with Moderna Therapeutics' Institutional Animal Care and Use guidelines. mRNA–LNP formulations diluted in Dulbecco's phosphate-buffered saline (PBS) were administered intravenously via the tail vein at a dose volume of 5 mL kg^{-1} using 29-gauge, 0.3 mL insulin syringes (BD Biosciences) following gentle warming under a heat lamp for approximately 3 min. Blood samples were collected (3 h, 6 h, and 24 h) via Submandibular vein and allowed to clot at room temperature in serum separator tubes (Greiner bio-one Cat # 450472). The tubes were centrifuged at 7000 rpm for 7 min, and serum samples were aliquoted and stored at -80 °C until analysis. Human erythropoietin (hEPO) concentrations were quantified using a commercially available hEPO ELISA kit (STEMCELL Technologies, Cat. #01630) following the manufacturer's instructions. The animals were euthanized *via* CO_2 asphyxiation, in accordance to Guideline GLD-04 (Guideline for euthanasia in rodents using Carbon Dioxide) the left lateral lobe was collected, weighed, and immediately transferred into 15cc conical tubes (Corning Cat # 430791) submerged in liquid nitrogen, once frozen samples were stored and shipped to Charles River Laboratories at -80 °C. Charles River Laboratories conducted lipid quantification analysis. Liver samples were homogenized using an Omni handheld homogenizer at a 19:1 volume (mL) to weight (g) ratio (H_2O:liver), resulting in a homogenization dilution factor of 20. Analytes were spiked into blank liver homogenates to prepare calibration standards. Sample extraction was performed by protein precipitation. Aliquots (50 µL) of sample homogenates, calibration standards, and blanks were transferred into a 96-well plate. Internal standard spike solution (arachidonoyl ethanolamide-d4, 400 µL, 50:50 ACN:IPA) was added to all samples except matrix

blanks, which received 400 µL of 50:50 ACN:IPA. The plate was covered, vortexed, and centrifuged for 5 min at >3000 rpm. Supernatant (200 µL) was transferred to a clean 96-well plate for analysis. Sample analysis was conducted using a Sciex 5500 triple quadrupole LC–MS/MS system equipped with an electrospray ionization source operating in positive ion mode. Chromatographic separation was achieved on a Higgins Analytical Clypeus C8 column (30 × 2.1 mm, 5 µm) using a gradient of 5 mM ammonium formate in 50:50:1 H_2O:MeOH:FA (mobile phase A) and 5 mM ammonium formate in 100:1 MeOH:FA (mobile phase B) at a flow rate of 1.2 mL/min. Analytes and internal standards were monitored by multiple reaction monitoring (MRM) of the respective [M+1]+ transition.

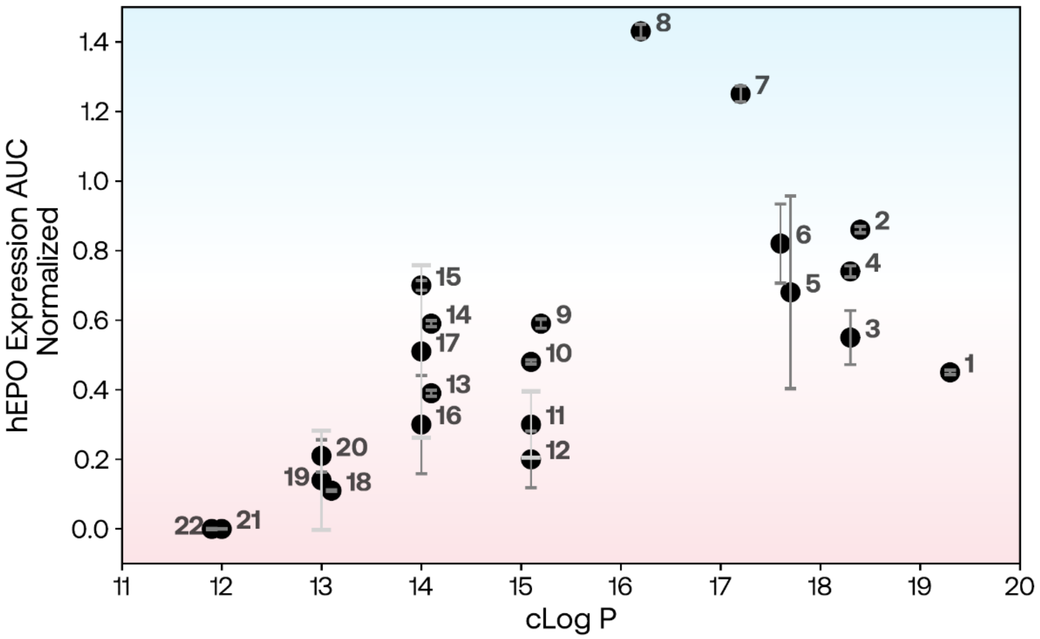

Figure S1. Normalized hEPO expression AUC of the LNPs formulated by ionizable lipids **1-22** and their cLogP values. Error bars represent the standard deviation in measurements from n=5 animal groups per lipid. The color spectrum is intended as a visual guide to show low (red) to high (blue) expression levels after normalization to **Lipid A**.

Table S2. *In vivo* mice hEPO expression and lipids remaining in liver (nmol/g) after 24h.

Lipids	Ratio of hEPO AUC to study control	log hEPO expression (6 h) to study control	Lipid remaining in liver 24h post dose (nmol/g)
**1**	0.45	0.94	140.54
**2**	0.86	0.99	57.95
**3**	0.55	0.96	65.42
**4**	0.74	0.98	65.78

**5**	0.68	0.98	34.58
**6**	0.82	0.99	39.87
**7**	1.25	1.01	27.31
**8**	1.43	1.03	25.33
**9**	0.59	0.96	11.94
**10**	0.48	0.95	12.36
**11**	0.30	0.95	8.53
**12**	0.20	0.89	8.89
**13**	0.39	0.94	4.4
**14**	0.59	0.97	2.64
**15**	0.70	0.98	15.24
**16**	0.30	0.92	5.12
**17**	0.51	0.96	4.91
**18**	0.11	0.85	0.27
**19**	0.14	0.87	1.82
**20**	0.21	0.90	0.95
**21**	0.00	0.42	0.03
**22**	0.00	0.65	0.11

References

[1] M. Cornebise, E. Narayanan, Y. Xia, E. Acosta, L. Ci, H. Koch, J. Milton, S. Sabnis, T. Salerno, K. E. Benenato, *Adv. Funct. Mater.* **2022**, *32*.

[2] S. Sabnis, E. S. Kumarasinghe, T. Salerno, C. Mihai, T. Ketova, J. J. Senn, A. Lynn, A. Bulychev, I. McFadyen, J. Chan, Ö. Almarsson, M. G. Stanton, K. E. Benenato, *Mol. Ther.* **2018**, *26*, 1509.
